# Performance of anterior segment OCT-based algorithms in the opportunistic screening for primary angle-closure disease

**DOI:** 10.1016/j.heliyon.2024.e28885

**Published:** 2024-03-30

**Authors:** Dapeng Mou, Jin Wang, Yue Wang, Xin Tang, Zhe Dong, Ningli Wang, Ye Zhang

**Affiliations:** a Beijing Tongren Eye Center, Beijing Key Laboratory of Ophthalmology and Visual Science, Beijing Tongren Hospital, Capital Medical University, Beijing, China; b Beijing Institute of Ophthalmology, Beijing, China

**Keywords:** Angle closure, Opportunistic screening, Algorithms, Anterior segment OCT

## Abstract

**Purpose:**

This study aimed to investigate the performance of deep learning algorithms in the opportunistic screening for primary angle-closure disease (PACD) using combined anterior segment parameters.

**Methods:**

This was an observational, cross-sectional hospital-based study. Patients with PACD and healthy controls who underwent comprehensive eye examinations, including gonioscopy and anterior segment optical coherence tomography (ASOCT) examinations under both light and dark conditions, were consecutively enrolled from the Department of Ophthalmology at the Beijing Tongren Hospital between November 2020 and June 2022. The anterior chamber, anterior chamber angle, iris, and lens parameters were assessed using ASOCT. To build the prediction models, backward logistic regression was utilized to select the variables to discriminate patients with PACD from normal participants, and the area under the receiver operating characteristic curve was used to evaluate the efficacy of the opportunistic screening.

**Results:**

The data from 199 patients (199 eyes) were included in the final analysis and divided into two groups: PACD (109 eyes) and controls (90 eyes). Angle opening distance at 500 μm, anterior chamber area, and iris curvature measured in the light condition were included in the final prediction models. The area under the receiver operating characteristic curve was 0.968, with a sensitivity of 91.74 % and a specificity of 91.11 %.

**Conclusion:**

ASOCT-based algorithms showed excellent diagnostic performance in the opportunistic screening for PACD. These results provide a promising basis for future research on the development of an angle-closure probability scoring system for PACD screening.

## Highlights

• Novel algorithm to detect primary angle-closure disease.
• Excellent diagnostic performance with 91.74 % sensitivity and 91.11 % specificity.
• Potentially useful tool for developing an angle-closure probability scoring system from opportunistic screening.

## 1 Introduction

Primary angle-closure disease (PACD) is responsible for a substantial proportion of cases of irreversible visual impairment and blindness in Asia, especially in China^1,2.^ It defined as appositional or synechial closure of the anterior chamber angle with or without symptoms and patients with PACD are usually diagnosed through routine eye examinations [3]. According to the definitions developed by the International Society for Geographical and Epidemiological Ophthalmology, PACD can be classified as primary angle-closure suspect (PACS), primary angle closure (PAC), or primary angle-closure glaucoma (PACG) [4]. In China, a total of 10.1 million people were estimated to be affected by PACG in 2020, and approximately 6.9 million people are currently suffering from PACG, causing blindness in one or both the eyes [1,2].

Early screening is most effective for diseases with serious consequences and long pre-symptomatic phases and thus can help in the prevention of the disease [4]. Preventive interventions, including medication and laser surgery at earlier PACD stages, such as PACS and PAC, could effectively reduce the risk of progression to the severe symptomatic stage with irreversible visual impairment [[6], [7], [8]].

A screening algorithm developed to meet the needs of a population- or community-based screening with a group of individuals at risk should have high specificity and sensitivity [9,10]. However, based on the findings of one of our previous studies, none of the existing methods achieved the combination of specificity and sensitivity needed for population-based screening of PACD [11]. Therefore, the opportunistic screening (also known as case detection) at clinics (screening those already attending health services for other reasons) seems more appropriate for PACD, especially in developing countries [10,12,13].

**Fig. 1:**
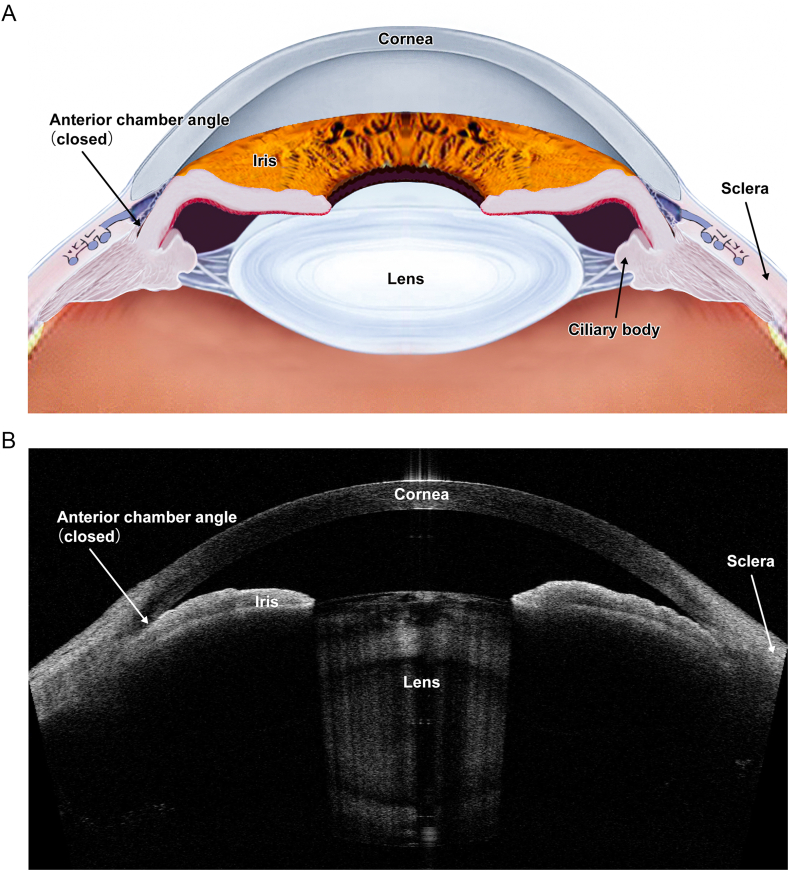
**Diagrammatic illustrated the pathogenetic characteristics of primary angle closure disease.** Panel A illustrated the pathogenesis of the primary angle-closure disease; Panel B illustrated angle closure on an anterior-segment OCT image.

The pathogenesis of PACD is characterized by mechanical or anatomical angle closure, resulting in elevated intraocular pressure (IOP) and subsequent glaucomatous optic neuropathy [14,15]. Hence, detecting the anterior segment anatomy, particularly the angle configuration, is the cardinal component for screening and diagnosing PACD [14,15]. [Fig. 1 (A, B)] Meanwhile, evidence from the literature suggests that angle closure occurs because of the anatomic and dynamic influences of various anterior segment structures in predisposed eyes [[16], [17], [18], [19]]. Our previous study demonstrated that using a combination of static and dynamic anterior segment optical coherence tomography (ASOCT) parameters could improve the screening efficacy for PACD at some level [20].

As far as we are concerned, only a limited number of studies focused on developing an algorithm based on ASOCT for opportunistic screening that includes both static and dynamic parameters. In this study, we sought to develop a machine-learning algorithm integrating both static and dynamic ASOCT parameters for detecting PACD through an opportunistic screening process.

## 2 Materials and methods

### 2.1 Study population and recruitment

This was an observational, cross-sectional hospital-based study. Patients with PACD and normal participants aged 40 years or older who underwent both gonioscopy and ASOCT imaging in light and dark conditions at the Department of Ophthalmology at the Beijing Tongren Hospital, between November 2020 and June 2022, were consecutively enrolled. The normal participants were defined as those attending the clinic with only a refractive error, dry eye, or other eye diseases that did not affect the study results, in addition to a history of IOP of <21 mmHg with open angles and a healthy optic nerve. The patients were excluded if they had any of the following: prior laser treatment or intraocular surgery, diagnosis of secondary angle-closure glaucoma, a history of trauma, congenital abnormalities, or ocular diseases other than cataract.

This study adhered to the tenets of the Declaration of Helsinki regarding research involving human participants. Written informed consent was obtained from all the participants, and the study was approved by the Ethics Committee of the Beijing Tongren Hospital (Approval No. TRECKY2019-120).

### 2.2 Study examination

Each participant underwent a though and standardized eye assessment, including presenting visual acuity and best-corrected visual acuity determined via a Snellen visual acuity chart, objective refraction assessed using a KR-8900 auto kerato-refractometer (Topcon, Tokyo, Japan), subjective refraction, slit-lamp examination, IOP measurement utilizing a Cannon TX-20 tonometer (Canon, Tokyo, Japan), gonioscopic examination, IOLMaster (V.5, Carl-Zeiss Meditec, Dublin, CA), and fundus examination conducted with a 90-D lens.

One of the two observers (J.W. and D.P.M.) conducted the dark-room indentation gonioscopy utilizing a one-mirror Goldmann lens (Ocular Instruments, Bellevue, WA) at a high magnification ( × 25). The specialists conducting the gonioscopy examination were unaware of the ASOCT findings. Static examination was conducted with a dim ambient illumination, using a shortened slit that avoided falling directly on the pupil. Following static gonioscopy, indentation gonioscopy was conducted with heightened illumination to identify any peripheral anterior synechiae. The two observers achieved a kappa value of 0.82 for evaluating the occludable angle in 30 eyes.

### 2.3 ASOCT image acquisition and analysis

The ASOCT scanner (CASIA2, Tomey Corporation, Nagoya, Japan) used a 1310-nm wavelength swept-source laser at a frequency of 0.3 s, obtaining 128 cross-sectional images evenly spaced 1.4° apart [21,22]. Prior to any contact procedure, each eye underwent imaging initially in dark conditions (approximately 3 lx to induce physiological mydriasis), and subsequently imaging in light conditions (approximately 200 lx). Patients were allowed to adapt to the dark for at least 3 min before the examination. The operator gently retracted the upper and lower eyelids to fully expose the testing position without applying additional pressure on the globe.

During ASOCT scanning, the patients were asked to focus on an internal fixation target with an auto-alignment function and were scanned using the lens biometry mode at one cross-sectional horizontal scan (nasal-temporal angles at 0–180°) for each patient. For each image, the SS and angle recess were manually corrected by a single-blind experienced observer (Y.Z.). SS was defined as the inward protrusion of the sclera where a change in the curvature of the corneoscleral junction was detected (Sakata et al., 2008). If the image quality was poor, then the imaging process was repeated.

**Fig. 2:**
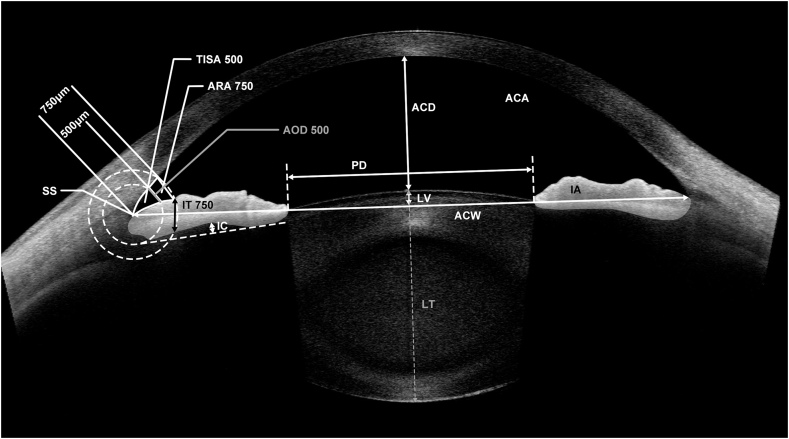
The parameters measured by the built-in semiautomated software in anterior-segment OCT images.

Once the locations of the two SS were determined, the following anterior chamber, angle, iris, and lens configuration parameters were measured by the built-in semiautomated software: angle opening distance at 500 μm from the SS (AOD500), trabecular-iris space area at 500 μm from the SS (TISA500), angle recess area at 750 μm from the SS (ARA750), ACA, ACV, ACW, ACD, iris thickness at 750 μm from the SS (IT750), IC, IA, IV, LV, lens thickness, and PD (Fig. 2). The definitions of AOD500, TISA 500, ARA750, ACA, ACV, ACW, IT750, IC, LV, PD, and IA have been reported previously [22,23]. Eyes with a PD increase of <0.5 mm after a physiological pupil dilation were excluded from the analysis. The change in IA was calculated by subtracting the IA in light conditions from the IA in the dark. Similarly, the change in PD was calculated as the PD in the dark minus the PD in light conditions.

### 2.4 Statistical analysis

The data from the right eye were included in the analysis only if the right or both the eyes met the inclusion and exclusion criteria, and the data from the left eye were included in the analysis only if the left eye qualified. The normality of continuous data was evaluated using the one-sample Kolmogorov–Smirnov test. Normally distributed continuous data are presented as means and standard deviations (SD), while non-normally distributed numerical data as medians and interquartile ranges. Categorical data are presented as frequencies and percentages. The two-sample independent *t*-test and Mann–Whitney *U* test were used to compare continuous data between patients with PACD and normal controls for normally and non-normally distributed data, respectively; the χ^2^ test was used to assess the differences in the categorical data. All tests were two-sided; Cohen's d term was used to calculate effect size, and its 95% confidence interval is also illustrated in this article. d is calculated by taking the difference in means of two samples divided by the SD of the samples. Cohen determined d values of 0.2, 0.5, and 0.8 indicate small, medium, and large effect sizes [24]. Statistical significance was defined as a P-value <0.05.

Univariate linear regression analyses were conducted to include parameters with a P-value <0.05. BLR was used to finalize the predictive model [25]. In BLR, parameters with the least significant that failed to meet the level for retaining in the model was removed, and remained excluded. This process was repeated until no other parameters met the specified removal levels [26]. Fourteen candidate static parameters under each condition were assessed separately. Three calculated parameters of changes, including IA, IV, and PD change from the dark to light conditions, were included for assessment, together with the parameters under each condition in the prediction models.

To prevent apparent correlations among the independent variables, AOD500 and ACA were chosen as representatives of the angle and anterior chamber dimensions, respectively. Nagelkerke's R^2^ was used to evaluate the best models for fitting the data. The best predictive model was evaluated using the area under the receiver operating characteristic curve, sensitivity, and specificity.

The Statistical Package for the Social Sciences (SPSS version 17.0 IBM, Armonk, NY) was used for data analysis. The estimates of the AUC along with the 95 % confidence interval (CI), sensitivity, and specificity were analysed utilizing MedCalc (version 20.218, MedCalc, Ostend, Belgium).

## 3 Results

A total of 238 patients (238 eyes) who underwent ocular examination and ASOCT measurements in light and dark conditions were included in the study. Thirty-nine eyes were excluded because of the following reasons: 17 eyes (7.1 %) with unidentified scleral spur (SS) and 22 eyes (9.2 %) with a pupil diameter (PD) change of less than 0.5 mm from the dark to light condition. The data from the remaining 199 patients (199 eyes) were analysed.

The mean age was 65.64 ± 8.96 years, and 127 patients (63.8 %) were women. A total of 109 (54.8 %) patients with PACD and 90 (45.2 %) healthy participants were included. Comparisons of the demographic characteristics and ocular biometric data between patients with PACD and healthy controls are shown in Table 1. Compared to healthy participants, those with PACD were older (P < 0.001), had worse presenting visual acuity (P < 0.001), thicker central cornea (P = 0.013), shallower anterior chamber depth (ACD; P < 0.001), and shorter axial length (P < 0.001). There were no significant differences in the proportion of sex or vertical cup-disc ratio between the two groups.

**Table 1:** Demographic data and ocular biometric measurements in patients with PACD and normal participants.

Parameter	Normal participants (*n* = 90)	Patients with PACD (*n* = 119)	Cohen's d (95% CI)	*P* value
Age (SD), years	62.3 (8.4)	68.4 (8.5)	−0.715 (−1.00, −0.426)	﹤0.001‡
Male (%)	32 (35.6)	40 (36.7)	–	0.868*
Female (%)	58 (64.4)	69 (63.3)		
PVA (IR)	0.61 (0.30, 1.00)	0.40 (0.19, 0.70)	–	﹤0.001^a^
IOP (IR), mmHg	13.1 (12.0, 15.0)	15.0 (12.7, 21.2)	–	﹤0.001^a^
VCDR (IR)	0.40 (0.30, 0.50)	0.40 (0.30, 0.60)	–	0.931^a^
CCT (SD), mm	511 (34)	524 (37)	−0.363 (−0.649, −0.076)	0.013‡
Central ACD (SD), mm	3.16 (0.39)	2.42 (0.32)	2.11 (1.761, 2.459)	﹤0.001‡
AL (IR), mm	23.37 (22.58, 24.50)	22.47 (22.07, 23.11)	–	﹤0.001^a^

PACD, primary angle-closure disease; CI, confidence interval; PVA, presenting visual acuity; IOP, intraocular pressure; VCDR, vertical cup-disc ratio; CCT, central corneal thickness; ACD, anterior chamber depth; AL, axial length; SD, standard deviation; IR, interquartile range.

a Mann–Whitney *U* test ‡ Two sample *t*-test.﹡χ2 test.

ASOCT parameters obtained under light and dark conditions and related changes in iris cross-sectional area (IA), iris volume (IV), and PD of patients with PACD and normal participants are summarized in Supplementary Tables 1 and 2 Compared with normal participants, patients with PACD had smaller AOD500 (P < 0.001), TISA500 (P < 0.001), ARA750 (P < 0.001), anterior chamber width (ACW; P < 0.001), anterior chamber area (ACA; P < 0.001), and anterior chamber volume (ACV; P < 0.001); shallower ACD (P < 0.001); larger IT750 (P < 0.001 under the light condition and P = 0.017 under the dark condition), iris curvature (IC; P < 0.001), LT (P < 0.001), and LV (P < 0.001) under the light and dark conditions; larger IA (P = 0.003) and PD (P = 0.006) under the dark condition; and smaller IA change (P < 0.001), PD change (P = 0.001), and IV change (P = 0.017) from the dark to light condition. There was no significant difference between patients with PACD and normal participants in IA and PD under the light condition, or in IV under the light and dark conditions.

Table 2, Table 3 illustrate the selection process of variables using univariate and multivariate logistic regression analyses under the light and dark conditions, respectively. The logistic model with a combination of parameters measured under the light condition showed the best fit, with a higher R^2^ (0.790 in the dark condition and 0.809 in the light condition). Backward logistic regression (BLR) was employed to incorporate the following three variables into the prediction models: AOD500 under the light condition (P < 0.001), ACA under the light condition (P = 0.019), and IC under the light condition (P = 0.041). The dynamic parameters were not included in the final model.

**Table 2:** Univariate and multivariate logistic regression analyses for variable selection using ASOCT parameters measured under the dark condition.

Variable	Univariate logistic regression			Multivariate logistic regression			
	OR (95 % CI)	P value	Nagelkerke R^2^	Beta	OR (95 % CI)	P value	Nagelkerke R^2^
AOD500 (μm)	0.976 (0.969, 0.983)	﹤0.001	0.731	−0.017	0.983 (0.972, 0.994)	0.002	0.790
ACA (mm^2^)	0.420 (0.322, 0.548)	﹤0.001	0.731	−0.539	0.584 (0.414, 0.822)	0.002	
IT750 (0.1 mm)	1.725 (1.097, 2.714)	0.018	0.038	–	–	–	
IC (0.1 mm)	2.311 (1.661, 3.214)	﹤0.001	0.206	−0.766	0.465 (0.186, 1.163)	0.102	
IA (mm^2^)	2.829 (1.388, 5.764)	0.004	0.057	–	–	–	
IV (mm^3^)	1.000 (0.941, 1.062)	0.994	﹤0.001				
LT (mm)	27.566 (9.937, 76.464)	﹤0.001	0.428	–	–	–	
LV (0.1 mm)	1.860 (1.565, 2.211)	﹤0.001	0.591	–	–	–	
PD (mm)	0.601 (0.416, 0.869)	0.007	0.052	–	–	–	
IA change (mm^2^)	0.042 (0.008, 0.230)	﹤0.001	0.099	–	–	–	
IV change (mm^3^)	0.786 (0.608, 1.016)	0.066	0.025				
PD change (mm)	0.278 (0.152, 0.510)	﹤0.001	0.128	–	–	–	

PACD, primary angle-closure disease; OR, odds ratio; CI, confidence interval; AOD500, angle opening distance at 500 μm; TISA500, trabecular-iris space at 500 μm; ARA750, angle recess area at 750 μm; ACD, anterior chamber depth; ACW, anterior chamber width; ACA, anterior chamber area; ACV, anterior chamber volume; IT750, iris thickness at 750 μm; IC, iris curvature; IA, iris cross-sectional area; IV, iris volume; LV, lens vault; PD, pupil diameter.

**Table 3:** Univariate and multivariate logistic regression analyses for variable selection using ASOCT parameters measured under the light condition.

Variable	Univariate logistic regression			Multivariate logistic regression			
	OR (95 % CI)	P value	Nagelkerke R^2^	Beta	OR (95 % CI)	P value	Nagelkerke R^2^
AOD500 (μm)	0.976 (0.970, 0.983)	﹤0.001	**0.742**	−0.023	0.977 (0.966, 0.988)	﹤0.001	0.809
ACA (mm^2^)	0.413 (0.317, 0.538)	﹤0.001	**0.733**	−0.371	0.690 (0.507, 0.940)	0.019	
IT750 (0.1 mm)	2.382 (1.476, 3.843)	﹤0.001	0.090	–	–	–	
IC (0.1 mm)	2.232 (1.619, 3.078)	﹤0.001	0.203	−0.955	0.385 (0.154, 0.961)	0.041	
IA (mm^2^)	1.446 (0.759, 2.754)	0.262	0.008				
IV (mm^3^)	0.983 (0.925, 1.044)	0.570	0.002				
LT (mm)	24.629 (9.483, 63.965)	﹤0.001	0.412	–	–	–	
LV (0.1 mm)	1.865 (1.569, 2.218)	﹤0.001	0.596	–	–	–	
PD (mm)	1.033 (0.706, 1.511)	0.868	﹤0.001				
IA change (mm^2^)	0.042 (0.008, 0.230)	﹤0.001	0.099	–	–	–	
IV change (mm^3^)	0.786 (0.608, 1.016)	0.066	0.025				
PD change (mm)	0.278 (0.152, 0.510)	﹤0.001	0.128	–	–	–	

PACD, primary angle-closure disease; OR, odds ratio; CI, confidence interval; AOD500, angle opening distance at 500 μm; TISA500, trabecular-iris space at 500 μm; ARA750, angle recess area at 750 μm; ACD, anterior chamber depth; ACW, anterior chamber width; ACA, anterior chamber area; ACV, anterior chamber volume; IT750, iris thickness at 750 μm; IC, iris curvature; IA, iris cross-sectional area; IV, iris volume; LV, lens vault; PD, pupil diameter.

**Fig. 3:**
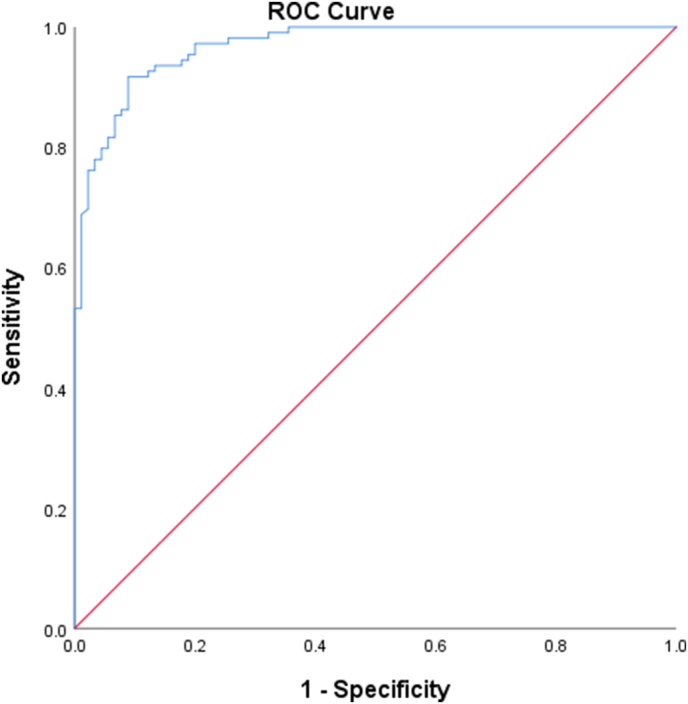
Receiver operating characteristic curve of the machine-learning algorithm.

Fig. 3 shows the prediction accuracy of the best BLR model. The area under the receiver operating characteristic curve (AUC) was 0.968 (95 % confidence interval [CI], 0.948–0.988) with a sensitivity of 91.74 (95 % CI, 84.90–96.20) and a specificity of 91.11 (95 % CI, 83.20–96.10).

## 4 Discussion

The combination of AOD500, ACA, and IC measured by ASOCT under the light condition had an excellent diagnostic performance in distinguishing PACD eyes from control eyes in opportunistic screening, with a sensitivity of 91.74 % and a specificity of 91.11 %.

PACD meets the classical criteria for disease screening. It can cause irreversible blindness, and affected patients may not seek medical care until significant visual field loss occurs, except for those with an acute attack [5,27]. Most importantly, definitive intervention methods are available to treat the disease and prevent visual impairment [6,7]. There are two main approaches to screening: the population-based approach and case detection (opportunistic screening) [10]. A population-based screening method should have at least 95–98% specificity with mid-80% sensitivity [9,10]. Four non-contact screening tests were used to detect PACS in a population that was evaluated in our previous study [11]. The results showed that none of the tests met the requirements for the population-based screening method. Subsequently, we attempted to establish algorithms that included static and dynamic ASOCT parameters for the identification of PACS. The results also showed that neither BLR, naïve Bayes’ classification, nor a neural network were suitable for population-based screening [20]. Therefore, we conducted this study to evaluate the performance of deep learning algorithms using static and dynamic ASOCT parameters in opportunistic screening for PACD.

The objective of opportunistic screening is to optimize the efficiency of deciding which patients should undergo gonioscopy to further determine the extent of peripheral anterior synechiae and severity of the disease. To provide definitive care, it is important to minimize missing PACD cases; therefore, we optimized our model for high sensitivity [10].

Several diagnostic methods can be used to detect the angle configuration in patients with PACD [11,23,28]. Gonioscopy is considered the definitive reference standard for identifying individuals at risk of angle closure. However, this contact-based examination is technically challenging, uncomfortable for patients, and dependent on a single-observer interpretation with moderate reproducibility [23,28]. ASOCT is a non-contact qualitative method for objectively detecting anterior segment landmarks and parameters in a comfortable manner for patients [29]. Hence, the strategy developed by ASOCT, which provides non-contact in vivo imaging of the anterior segment of the eye, should be appropriate, acceptable, cost-effective, and reasonable for opportunistic screening. In addition, it can assess the unique anterior segment characteristics, such as iris thickness, IC, LV, and ACA, under dynamic changes from light to dark conditions, which are otherwise impossible to assess quantitatively by slit-lamp examination or by gonioscopy alone [17,18,[29], [30], [31]].

Several studies have used ASOCT to detect PACD in healthy controls of community-based populations. Nongpiur et al. established a stepwise logistic regression model with six ASOCT parameters to diagnose angle closure 95 % of the time [32]. Tan et al. demonstrated that the combined parameters of ACA, LV, and IC derived from the horizontal meridian scans could achieve an AUC of 0.88 in detecting angle closure [33]. The variations in predictive efficacy between our study and others may stem from differences in patient demographics, disease severity, angle-closure mechanisms involved, and the selection of variables used for constructing the models.

Ma et al. used the mean ACD with a cut-off of 2.2 mm to distinguish patients with PACD from healthy controls in a hospital-based study, with an AUC of 0.94 and sensitivity and specificity of 90.2 % and 85.2 %, respectively, and evaluated the diagnostic performance in differentiating PAC/PACG eyes from PACS eyes [34]. The use of a single parameter may limit its use in Asian populations, in which non-pupillary block and mixed angle closure mechanisms account for most cases [16,19,28]. Our study included three ASCOT parameters under light conditions in the final model, which could be easily obtained from a single axial eye scan to evaluate the angle configuration more precisely. We also considered that it is sufficient to screen PACD from healthy controls rather than subdividing the disease into stages, such as PACS, PAC, or PACG.

Compared to IOP, which is the main parameter in the early development of screening tests for glaucoma, structural or functional abnormality diagnostic tests are now widely used [20,[32], [33], [34], [35]]. In PACD, diagnostic tests based on structural changes in the anterior segment are more appropriate. Based on our prior study, the development of PACD is influenced not solely by static anatomical factors but also by the dynamic reactions of the iris under light and dark conditions [19,20,36]. In this study, we included parameters under both conditions; however, the final model excluded the parameters of dynamic iris changes. Therefore, we speculated that the patients included in the opportunistic screening had more severe diseases compared to those in the population-based study we previously conducted, and that dynamic iris change might act as an angle-closure mechanism at the early stage of the disease.

As far as we know, this study marks the first attempt to establish a novel algorithm aimed at detecting PACD in healthy controls by combining anterior segment parameters with opportunistic screening. We used the upgraded ASOCT, which could rapidly image all 360° of the anterior segment and extract much more data than traditional ASOCT [21,22]. However, this study had few limitations. First, we utilized only one meridional scan and did not use multiple scans of various meridians to determine whether the accuracy of the algorithm could be improved. However, Tan et al. showed that horizontal scanning had the best performance and adding all eight frames reduced the diagnostic accuracy to 63 % [33]. Second, the ASOCT software was semiautomatic, and the SS and angle recess positions needed to be adjusted manually, which could have resulted in subjectivity at some level [37]. Third, all participants were recruited from a single hospital in China and may have induced a sampling bias. Fourth, our study comprised participants of Chinese ethnicity; hence, the findings may not be directly applicable to other ethnic populations. And finally, the results of the algorithm only showed great sensitivity and specificity in a clinical-based populations, further population-based study should be conducted to further test its efficacy.

In conclusion, our study demonstrated an excellent diagnostic performance in distinguishing PACD eyes from normal eyes through opportunistic screening, with 91.74 % sensitivity and 91.11 % specificity. These parameters make it a potentially useful tool for clinical use and may serve as the basis for future research on developing an angle-closure probability scoring system assisted by artificial intelligence technology for angle-closure disease screening.

## Ethics statement

The study was approved by the Ethics Committee of the 10.13039/100022815Beijing Tongren Hospital (Approval No. TRECKY2019-120), and written informed consent was obtained from all the participants.

## Patient consent for publication

Written informed consent was obtained from all the participants.

## Funding

The study was supported by the 10.13039/100014717National Natural Science Foundation for Young Scientists of China (Grant Number 81900847), the General Program of 10.13039/501100001809National Natural Science Foundation of China (Grant Number 82371050), the 10.13039/100016126Beijing Hospitals Authority Youth Program (Grant Number QML20210201) and the 10.13039/501100001809National Natural Science Foundation of China (Grant Number 62020106015). The funding organizations had no role in the design or conduct of the study.

## Data sharing statement

The authors declare that all data supporting he findings of this study are available within the paper. They are not publicly available, and restrictions apply to their use. All requests would require evaluation on an individual basis and can be made by contacting wningli@vip.163.com.

## CRediT authorship contribution statement

**Dapeng Mou:** Writing – original draft, Methodology, Conceptualization. **Jin Wang:** Writing – original draft, Methodology, Investigation, Formal analysis. **Yue Wang:** Resources, Investigation, Formal analysis. **Xin Tang:** Resources, Methodology, Investigation, Data curation. **Zhe Dong:** Resources, Methodology, Investigation. **Ningli Wang:** Writing – review & editing, Supervision, Project administration, Conceptualization. **Ye Zhang:** Writing – review & editing, Writing – original draft, Supervision, Project administration, Methodology, Funding acquisition, Conceptualization.

## Declaration of competing interest

The authors declare that they have no known competing financial interests or personal relationships that could have appeared to influence the work reported in this paper.
