# Supplementary material for: Performance of anterior segment OCT-based algorithms in the opportunistic screening for primary angle-closure disease
Source: Heliyon. 2024 Mar 30;10(7):e28885. doi: 10.1016/j.heliyon.2024.e28885 (PMC11002240; doi:10.1016/j.heliyon.2024.e28885)
Supplement: Multimedia component 1 [file mmc1.docx]

**Supplement Table 1. Anterior Chamber, Angle, Lens, and Iris Parameters Measured by ASOCT in Light and Dark Conditions in PACD and Normal Subjects**

| **Conditions** | **Parameter** | **Normal subjects (*n* = 91)** | **PACD subjects (*n* = 110)** | **Cohen’s d (95%CI)** | ***P* value*** |
| --- | --- | --- | --- | --- | --- |
| **Light** | AOD500 (IR), mm | 0.392 (0.339, 0.553) | 0.187 (0.128, 0.233) | ---- | ﹤0.001† |
|  | TISA500 (IR), mm^2^ | 0.148 (0.125, 0.188) | 0.080 (0.050, 0.106) | ---- | ﹤0.001† |
|  | ARA750 (IR), mm^2^ | 0.284 (0.246, 0.377) | 0.150 (0.106, 0.191) | ---- | ﹤0.001† |
|  | ACD (IR), mm | 2.669 (2.456, 3.024) | 1.952 (1.760, 2.195) | ---- | ﹤0.001† |
|  | ACW (SD), mm | 11.328 (0.428) | 11.105 (0.406) | 0.535（0.250，0.818） | ﹤0.001‡ |
|  | ACA (IR), mm^2^ | 19.454 (17.658, 22.736) | 13.460 (11.721, 15.648) | ---- | ﹤0.001† |
|  | ACV (IR), mm^3^ | 128.532 (112.407, 152.287) | 81.488 (67.883, 99.297) | ---- | ﹤0.001† |
|  | IT750 (SD), mm | 0.352 (0.062) | 0.386 (0.063) | -0.539（-0.823，-0.253） | ﹤0.001‡ |
|  | IC (IR), mm | 0.141 (0.027, 0.191) | 0.207 (0.125, 0.264) | ---- | ﹤0.001† |
|  | IA (IR), mm^2^ | 3.120 (2.705, 3.429) | 3.172 (2.890, 3.483) | ---- | 0.352† |
|  | IV (SD), mm^3^ | 35.401 (4.885) | 35.021 (4.528) | 0.081（-0.200，0.362） | 0.572‡ |
|  | LT (SD), mm | 4.370 (0.464) | 4.941 (0.399) | -1.330（-1.638，-1.019） | ﹤0.001‡ |
|  | LV (SD), mm | 0.228 (0.328) | 0.783 (0.303) | -1.764（-2.091，-1.433） | ﹤0.001‡ |
|  | PD (SD), mm | 3.818 (0.723) | 3.836 (0.749) | -0.023（-0.303，0.256） | 0.869‡ |
| **Dark** | AOD500 (IR), mm | 0.391 (0.292, 0.525) | 0.174 (0.105, 0.206) | ---- | ﹤0.001† |
|  | TISA500 (IR), mm^2^ | 0.144 (0.113, 0.185) | 0.067 (0.045, 0.091) | ---- | ﹤0.001† |
|  | ARA750 (IR), mm^2^ | 0.278 (0.224, 0.363) | 0.131 (0.090, 0.174) | ---- | ﹤0.001† |
|  | ACD (IR), mm | 2.664 (2.460, 3.011) | 1.961 (1.759, 2.195) | ---- | ﹤0.001† |
|  | ACW (SD), mm | 11.324 (0.394) | 11.108 (0.405) | 0.539（0.254，0.822） | ﹤0.001‡ |
|  | ACA (IR), mm^2^ | 19.993 (17.980, 23.324) | 13.839 (11.957, 16.270) | ---- | ﹤0.001† |
|  | ACV (IR), mm^3^ | 132.144 (116.246, 160.724) | 84.085 (68.104, 104.028) | ---- | ﹤0.001† |
|  | IT750 (SD), mm | 0.386 (0.064) | 0.408 (0.063) | -0.344（-0.624，-0.062） | 0.017‡ |
|  | IC (IR), mm | 0.139 (0.019, 0.199) | 0.203 (0.134, 0.255) | ---- | ﹤0.001† |
|  | IA (SD), mm^2^ | 2.807 (0.435) | 2.979 (0.384) | -0.422 （-0.704，-0.140） | 0.003‡ |
|  | IV (SD), mm^3^ | 34.360 (4.939) | 34.355 (4.386) | 0.001（-0.279，0.281） | 0.994‡ |
|  | LT (SD), mm | 4.355 (0.460) | 4.942 (0.407) | -1.358（-1.678，-1.034） | ﹤0.001‡ |
|  | LV (SD), mm | 0.230 (0.329) | 0.778 (0.296) | -1.762（-2.089，-1.431） | ﹤0.001‡ |
|  | PD (IR), mm | 4.929 (4.374, 5.606) | 4.753 (4.016, 5.066) | ---- | 0.006† |

AOD500, angle opening distance at 500 μm; TISA500, trabecular-iris space at 500 μm; ARA750, angle recess area at 750 μm; ACD, anterior chamber depth; ACW, anterior chamber width; ACA, anterior chamber area; ACV, anterior chamber volume; IT750, iris thickness at 750 μm; IC, iris curvature; IA, iris cross-sectional area; IV, iris volume; LV, lens vault; SD, standard deviation; IR, interquartile range.

† Mann-Whitney U test. ‡ Two sample t-test.

**Supplement Table 2. IA Changes and IV Changes Measured by ASOCT in Light and Dark Conditions in PACD and Normal Subjects**

| **Parameter** | **Normal subjects (*n* = 91)** | **PACD subjects (*n* = 110)** | **Cohen’s d (95%CI)** | ***P* value*** |
| --- | --- | --- | --- | --- |
| IA Change (SD), mm^2^ | 0.299 (0.191) | 0.196 (0.174) | 0.562（0.276，0.846） | ﹤0.001‡ |
| IV Change (IR), mm^3^ | 0.940 (0.443, 1.700) | 0.752 (0.102, 1.200) | ---- | 0.017† |
| PD Change (IR), mm | 1.085 (0.772, 1.420) | 0.610 (0.356, 1.045) | ---- | ﹤0.001† |

IA, iris cross-sectional area; IV, iris volume; pupil diameter; SD, standard deviation; IR, interquartile range.

† Mann-Whitney U test. ‡ Two sample t-test.
